# The gene expression profile of a drug metabolism system and signal transduction pathways in the liver of mice treated with *tert*-butylhydroquinone or 3-(3'-*tert*-butyl-4'-hydroxyphenyl)propylthiosulfonate of sodium

**DOI:** 10.1371/journal.pone.0176939

**Published:** 2017-05-03

**Authors:** Alexandra B. Shintyapina, Valentin A. Vavilin, Olga G. Safronova, Vyacheslav V. Lyakhovich

**Affiliations:** 1 Institute of Molecular Biology and Biophysics, Novosibirsk, Russia; 2 Novosibirsk State University, Novosibirsk, Russia; Alexandria University, EGYPT

## Abstract

*Tert*-butylhydroquinone (tBHQ) is a highly effective phenolic antioxidant used in edible oils and fats in foods as well as in medicines and cosmetics. TBHQ has been shown to have both chemoprotective and carcinogenic effects. Furthermore, it has potential anti-inflammatory, antiatherogenic, and neuroprotective activities. TBHQ induces phase II detoxification enzymes via the Keap1/Nrf2/ARE mechanism, which contributes to its chemopreventive functions. Nonetheless, there is growing evidence that biological effects of tBHQ may be mediated by Nrf2-independent mechanisms related to various signaling cascades. Here, we studied changes in gene expression of phase I, II, and III drug metabolizing enzymes/transporters as well as protein levels and activities of cytochromes P450 (CYPs) elicited by tBHQ and its structural homolog TS-13 in the mouse liver. Next, we carried out gene expression analysis to identify signal transduction pathways modulated by the antioxidants. Mice received 100 mg/kg tBHQ or TS-13 per day or only vehicle. The liver was collected at 12 hours and after 7 days of the treatment. Protein and total RNA were extracted. Gene expression was analyzed using Mouse Drug Metabolism and Signal Transduction PathwayFinder RT^2^Profiler^™^PCR Arrays. A western blot analysis was used to measure protein levels and a fluorometric assay was employed to study activities of CYPs. Genes that were affected more than 1.5-fold by tBHQ or TS-13 treatment compared with vehicle were identified. Analysis of the gene expression data revealed changes in various genes that are important for drug metabolism, cellular defense mechanisms, inflammation, apoptosis, and cell cycle regulation. Novel target genes were identified, including xenobiotic metabolism genes encoding CYPs, phase II/III drug metabolizing enzymes/transporters. For Cyp1a2 and Cyp2b, we observed an increase in protein levels and activities during tBHQ or TS-13 treatment. Changes were found in the gene expression regulated by NFκB, androgen, retinoic acid, PI3K/AKT, Wnt, Hedgehog and other pathways.

## Introduction

**Fig 1:**
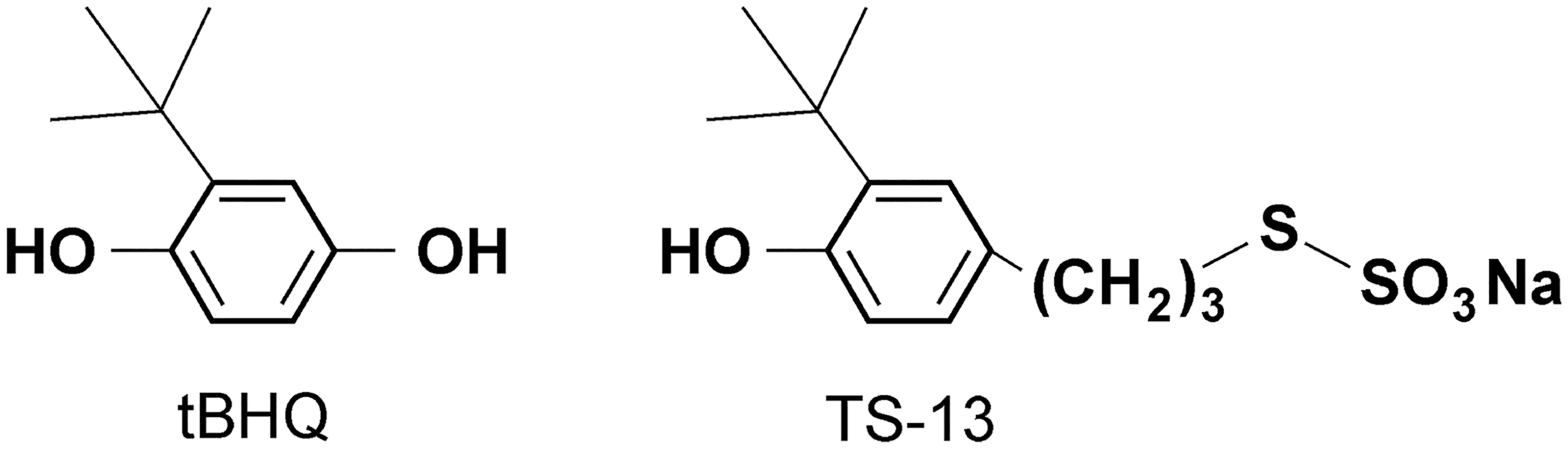
Chemical structures of tBHQ and TS-13.

*Tert*-butylhydroquinone (tBHQ; Fig 1) is a highly effective phenolic antioxidant, together with other tertiary butylated phenols (e.g., butylated hydroxyanisole (BHA), 2-t*ert*-Butyl-1,4-benzoquinone (TBQ), 2,5-Di-*tert*-butyl-1,4-benzoquinone (DTBBQ) belonging to a large family of synthetic phenolic antioxidants commonly used as food preservatives for unsaturated vegetable oils and many edible animal fats, fried foods, dried fish products, as well as in medicines and cosmetics [1, 2]. TBHQ has been shown to have both chemoprotective and carcinogenic effects [3], depending on a dose, duration of treatment, and a tissue type [4, 5]. A chemopreventive role of tBHQ is thought to be related mainly to the induction of phase II detoxification enzymes; this phenomenon has a potential tumor-preventing activity. These cellular responses accelerate detoxication of electrophiles and reactive oxygen species (ROS), and thereby protect cells from mutagenesis and neoplasia [6]. The main well-known mechanism of protection from ROS by tBHQ and its homologs is activation of the Kelch-like erythroid CNC homologue (ECH)-associated protein 1/Nuclear factor erythroid 2-related factor 2 (Keap1/Nrf2) signaling pathway via a covalent modification of cysteine SH-groups of Keap1. Nrf2 is quickly released from Keap1 (tBHQ causes Keap1 polyubiquitination and Nrf2 stabilization), relocates to the nucleus, and binds to an antioxidant response element (ARE), thus inducing phase II detoxification enzymes and production of antioxidant enzymes crucial for the cellular defense against electrophilic compounds and ROS [7].

Redox properties of synthetic chemicals are crucial for a Keap1/Nrf-2-mediated response of ARE-containing genes [8]. At the same time, the chemical structure of synthetic phenolic antioxidants, besides their redox properties, is important for their biological effects [9]. There is growing evidence that together with the classical mechanism, there are alternative cell targets for implementation of the biological effects of classical Nrf2 activators such as tBHQ, BHA, and other quinones [10–14].

Recently, a new *tert*-butylated phenol with a thiosulfonate group, 3-(3’-*tert*-butyl-4’-hydroxyphenyl)propylthiosulfonate of sodium (TS-13) was obtained (Fig 1) [15].

Earlier, we have shown that this compound is one of the most potent among derivatives orto-*tert*-butyl-phenols in terms of enhancement of gene expression of two major enzymes of the antioxidative defense system—Glutathione S-transferase pi 1 (*Gstp1)* and NAD(P)H:quinone oxidoreductase 1(*Nqo1*) via Nrf2/ARE activation [16]. It should be mentioned that TS-13, in contrast to tBHQ, is water soluble and thus has better bioavailability.

In the past, it was thought that tBHQ, BHA, and other quinones are monoactivators of phase II xenobiotic-metabolizing enzymes [17–19]. Later, it was shown that tBHQ can induce genes and proteins of phase I metabolizing enzymes, cytochromes P450 (CYP), such as Cyp1a1 [20, 21], CYP2C9, and CYP2C19 [22], and it was found that BHA also raises activity of Cyp2b10 [23]. The ability of tBHQ to interact with the aryl hydrocarbon receptor (AhR) as a ligand was demonstrated in cell culture (although with lesser affinity in comparison with AhR’s classical ligand 2,3,7,8-tetrachlorodibenzo-p-dioxin (TCDD), and thus to induce Cyp1a1 at the transcriptional level [20, 21]. However, authors of [21] emphasized that tBHQ can modulate mitogen-activated protein kinase (MAPK) signaling cascades, resulting in the phosphorylation of AhR and its activation. In addition, CYP2C9 is induced through activation of the binding site of transcription factor activator protein 1 (AP-1) in the promoter of *CYP2C9* [22]. The metabolism and especially bioactivation of carcinogens are dominated by CYP enzymes. Within this group, six CYPs—1A1, 1A2, 1B1, 2A6, 2E1, 2B and 3A4—account for 77% of the CYP activation reactions [24]

Thus, an increase in the activity of CYPs via mechanisms—independent of activation of xenobiotic-sensing nuclear receptors by tBHQ as a ligand or their phosphorylation by kinases—may cause changes in the balance of toxification/detoxification of various chemicals (including tBHQ) and explain tumor-promoting properties.

As for other biological effects of tBHQ, it has also been shown to have potential anti-inflammatory, atheroprotective, hepatoprotective, and neuroprotective activities, including protection against acute toxicity of chemicals and modulation of macromolecule synthesis and an immune response [11, 14, 25, 26]. In particular, some phenolic antioxidants, namely, classic phase II inducers, have an anti-inflammatory effect [9, 27, 28], which is thought to be mediated by activation of Nrf2 [29, 30], inhibition of nuclear factor κB (NFkB; a key regulator of immune and inflammatory responses) [27, 30, 31], and by influence on MAPKs engaged in complex crosstalk between Nrf2 and NFkB pathways [32]. The anti-inflammatory effect of TS-13 was shown on *in vivo* models of acute inflammation [33]. We have recently shown that the mechanism of this effect may be related to downregulation of p65 and p50 subunits of NFkB and reduced phosphorilation of activating transcription factor 2 (ATF-2) [34].

Thus, a combination of the Keap1/Nrf2/ARE-mediated mechanism and the interaction of phenolic antioxidants with other unknown targets in signal transduction pathways has pleiotropic effects of the phenolic antioxidants that drive responses in cells. Biological analysis of a cellular response to the chemicals may identify important aspects related to signal-induced antioxidative, anti-inflammatory, chemopreventive, and procarcinogenic mechanisms.

The present study was designed to investigate the influence of tBHQ and TS-13 on gene expression of phase I, II, and III xenobiotic-metabolizing enzymes/transporters, and on protein expression and activity of CYPs in BALB/c mice, and then to evaluate how tBHQ or TS-13 modulate the expression of genes related to various cell signaling cascades.

## Materials and methods

### Chemicals

The following chemicals were used: antioxidants: tBHQ (*tert*-butylhydroquinone) (Sigma-Aldrich), 3-(3'-*tert*-butyl-4'-hydroxyphenyl)propylthiosulfonate of sodium (TS-13) was synthesized as described previously [15]. The structures of the compounds are shown in Fig 1.

### Animals and experimental treatments

This study’s protocol was approved by the Local Committee on the Ethics of Animal Experiments of the Institute of Molecular Biology and Biophysics (Permit Number: 2/2012). BALB/c male mice (10 to 12 weeks old, average weight 20 ± 1.6 g) were housed in cages (at four animals per cage) with *ad libitum* access to feed and water. Corresponding phenolic antioxidants (tBHQ or TS-13) resuspended in a 2% starch gel were administered via gavages at a dose of 100 mg/kg b.w. per day. Animals of control groups were given only the 2% starch gel. Control and experimental groups consisted of 4–5 animals each. The animals of control groups and experimental groups were euthanized by cervical dislocation at 12 h or after 7 days of administration (12 h after last administration) of vehicle or the antioxidants. Liver specimens were taken and stored at –80°C until use.

### RNA extraction and cDNA synthesis

Pieces of murine livers (25–35 mg) were frozen in liquid nitrogen, and total RNA was extracted with the RNeasy Plus Mini Kit (Qiagen), including on-column DNase treatment to remove genomic DNA, following the manufacturer’s recommendations. The quality of RNA was tested on a 2% agarose gel. The A_260_/A_280_ ratio of RNA was 1.8–2.0. Concentration of RNA was measured using the Quant-iT RNA Assay Kit and a Qubit Fluorometer (Invitrogen). Next, 1 μg of RNA was reverse-transcribed using the ABI High Capacity RNA to cDNA kit (Applied Biosystems) according to the manufacturer's instructions.

### RT^2^Profiler PCR Arrays

cDNA samples from mice of the same group were pooled and analyzed for expression of 84 genes involved in mouse drug metabolism and 84 genes of signal transduction pathways using RT^2^ Profiler PCR Mouse Drug Metabolism Array (PAMM-002, Qiagen) and RT^2^ Profiler PCR Signal Transduction PathwayFinder PCR Array (PAMM-014, Qiagen), respectively. Briefly, cDNA template was combined with an instrument-specific and ready-to-use RT^2^ SYBR Green qPCR Master Mix (Qiagen). Equal aliquots of this mixture (25 μL) were added to each well of the same PCR array plate containing the predispensed gene-specific primer sets. PCR was conducted on a CFX96 Real-Time PCR Detection System (Bio-Rad). Each PCR was repeated three times. PCR Array quantification was based on the threshold cycle (C_t_) number. A gene was considered undetectable at C_t_ > 35, according to the RT^2^ Profiler PCR System user manual. Melt/dissociation curves were analyzed after each run to ensure single products. The RT^2^Profiler PCR Array software package (http://www.sabiosciences.com/pcr/arrayanalysis.php) was used for ΔΔC_t_–based fold change calculations from real-time PCR Array data.

### TaqMan real-time PCR

qPCR was performed using a CFX96 Real-Time PCR Detection System (Bio-Rad) to confirm PCR array results. We used predesigned TaqMan probes and primers for target genes *Nqo1*, *Cyp2b*, *Myc*, *Jun* (DNK-Sintez, Russia). Cy5-labeled *Gapdh* and *β-actin* premade TaqMan probes and primers (DNK-Sintez, Russia) served as the endogenous controls. Each sample was run in triplicate, and each reaction contained 250 nM target gene-specific TaqMan probes and primers, 250 nM reference gene-specific TaqMan probes and primers (*Gapdh* or β-actin), 12.5 μL of the 2x TaqMan Master mixture (Sintol, Russia), 5 μL of the 1:100 diluted cDNA sample, and was adjusted with double distilled H_2_O to final volume 25-μL. Efficiencies for the analyzed genes were 100% ± 10%. Fold changes values were determined by the 2^-ΔΔCt^ method.

### Isolation of liver microsomes

Mouse livers were perfused at 4°C with a solution of 20 mM Tris-HCl (pH 7.4), 1.15% KCl prior to homogenization. Liver microsomes were prepared by a standard method of differential centrifugation and sucrose gradient centrifugation in 20 mM Tris-HCl (pH 7.4) with 1.15% KCl [35]. The first extract from the homogenate was prepared by centrifugal fractionation at 10,000 x *g* for 20 min on a K-24D centrifuge (MLV ZentrifugenbauEngelsdorf). The supernatant was centrifuged at 106,000 x *g* for 60 min on ultracentrifuge VAC-602 (Janetzki). The pellet was resuspended in 0.1 M phosphate buffer (pH 7.4), containing 20% glycerin. All steps were carried out at 4°C. Microsomal protein concentration was measured as described elsewhere [36].

### Western blot

We used 30–50 μg of microsomal protein for western blots to measure CYP levels. Proteins were separated by polyacrylamide gel electrophoresis (SDS-PAGE) in a 10% gel, and transferred to a 0.45-μm polyvinylidene fluoride (PVDF) membrane, then the blot was blocked with 5% skim milk and 0.3% Tween 20 dissolved in phosphate-buffered saline (PBS). The membrane was washed in PBS three times for 10 min and incubated for 1.5 h at 37°C with primary rabbit anti-mouse antibodies for recognizing CYP1A1/2 and CYP2B (clone 14H5 and 12F10, respectively), which were obtained from Dr Grishanova [37] and diluted 1:20 in TBS. A rabbit anti-mouse β-actin primary antibody (diluted 1:500; Sigma-Aldrich, St. Louis, MO) was used to ensure equal loading of samples. Goat anti-rabbit IgG (Bio-Rad) alkaline-phosphatase-conjugated secondary antibodies were used to detect the primary antibodies to CYP1A1/2, CYP2B, and β-actin. The protein bands were visualized colorimetrically with alkaline-phosphatase fastTM Tablets (Sigma-Aldrich) as substrates, according to the manufacturer’s directions. Colorimetric blots were scanned on a Bio-Rad VersaDoc 4000 Imaging System (Bio-Rad), and the bands were quantified in the ImageJ software (NIH, Bethesda, MD, USA). The experiments were performed using equipment of center of “Proteomic analysis” of the Institute of Molecular Biology and Biophysics (Novosibirsk, Russia).

### Enzymatic activity assay

The activities of Cyp1a1, Cyp1a2, and Cyp2b10 were determined by a fluorometric assay [38] by monitoring the formation of enzymatic products of *O*-dealkylation of their selective substrates (7-ethoxy-, 7-methoxy-, and 7-penthoxy-resorufin, respectively). The reaction mix contained 0.2 ml of a buffer (50 mM HEPES, 15 mM MgCl_2_, 1 mM EDTA, pH 7.6), 10 or 20 μg of microsomal protein, 1 μM substrate and 1 mM NADPH. The formation of the enzymatic product, resorufin, was monitored continuously by fluorescence using an excitation wavelength of 530 nm and an emission wavelength of 580 nm on a plate reader EnVision (PerkinElmer, USA). Resorufin standards were used to calibrate the method. Enzymatic activity was expressed in pmol × min^-1^ × mg^-1^ protein.

### Statistical analysis

Data are representative of 4–5 animals per group and are expressed as mean ± standard deviation (SD). The differences between groups were analyzed by Student’s *t* test. Differences with p < 0.05 were considered significant.

## Results

**Fig 2:**
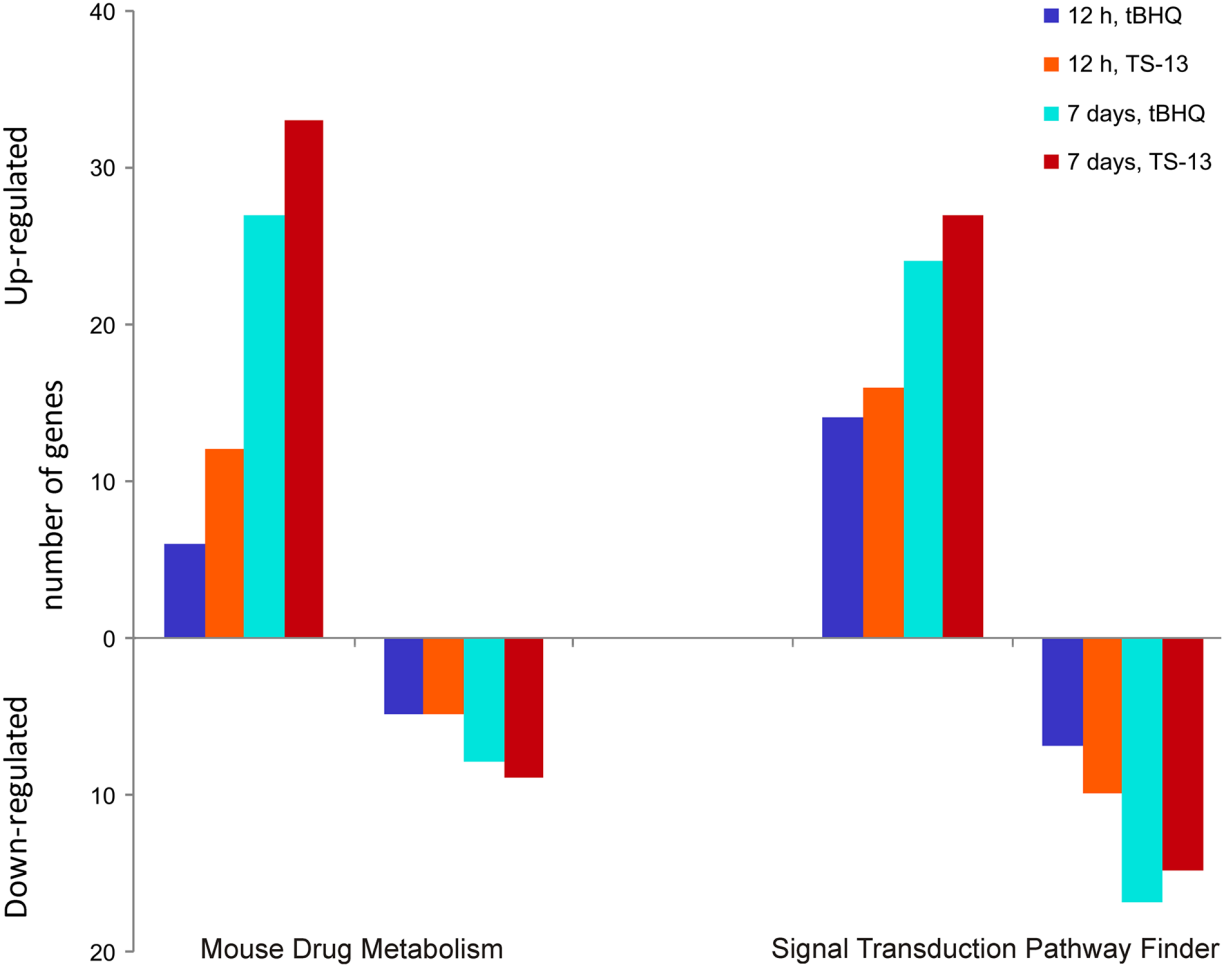
A summary of genes modulated (more than 1.5-fold) by tBHQ or TS-13 in the mouse liver. Hepatic gene expression patterns were analyzed after 12 h or 7 days of administration of tBHQ or TS-13.

RT^2^ PCR array tools were utilized to identify various biological pathways that are perturbed by exposure to tBHQ or its structural analog TS-13. The qRT-PCR Mouse drug metabolism array results showed that 5 of 84 genes at 12 h of tBHQ exposure were downregulated, while 6 genes were upregulated. After 12-h exposure to TS-13, we observed increased expression of 12 genes and decreased expression of 5. After 7 days of treatment of mice with tBHQ, the expression of 35 genes was changed (27 increased and 8 decreased) in the mouse liver. By comparison, treatment with TS-13 caused a change in the expression of 42 genes (33 increased and 9 decreased; Fig 2).

In general, the two compounds had similar effects on gene expression, showing up- or downregulation of the same genes (Table 1). However, in the case of TS-13, more genes were upregulated, and strength of the effect was greater as compared with tBHQ.

**Table 1:** Mouse drug metabolism PCR array.

Gene symbol	Description	tBHQ fold change		TS-13 fold change	
		12 h	7 d	12 h	7 d
**Drug Transporters:** Metallothioneins:					
*Mt2*	Metallothionein 2	1.6	-12.5	2.3	-25.1
*Mt3*	Metallothionein 3	1.7	-	1.7	-
P-Glycoprotein Family:.					
*Abcb1a*	ATP-binding cassette, sub-family B (MDR/TAP), member 1A	-	2.3	1.6	2.5
*Abcb1b*	ATP-binding cassette, sub-family B (MDR/TAP), member 1B	2.5	-	2.3	1.9
*Abcb4*	ATP-binding cassette, sub-family B (MDR/TAP), member 4	-	2.8	-	2.1
*Abcc1*	ATP-binding cassette, sub-family C (CFTR/MRP), member 1	-	**-**	-	-
*Gpi1*	Glucose phosphate isomerase 1	-	-	-	-
**Phase I Metabolizing Enzymes:** P450 Family:					
*Cyp11b2*	Cytochrome P450, family 11, subfamily b, polypeptide 2	-	1.8	-	2.1
*Cyp17a1*	Cytochrome P450, family 17, subfamily a, polypeptide 1	-	-4.3	-	-8.1
*Cyp19a1*	Cytochrome P450, family 19, subfamily a, polypeptide 1	-	-	-	-
*Cyp1a1*	Cytochrome P450, family 1, subfamily a, polypeptide 1	-	1.9	1.6	3.0
*Cyp1a2*	Cytochrome P450, family 1, subfamily a, polypeptide 2	-	2.0	-	2.6
*Cyp27b1*	Cytochrome P450, family 27, subfamily b, polypeptide 1	-	-	-	-
***Cyp2b10***	Cytochrome P450, family 2, subfamily b, polypeptide 10	3.2	2.2	2.1	2.0
*Cyp2c29*	Cytochrome P450, family 2, subfamily c, polypeptide 29	-	1.8	1.5	4.0
*Cyp2e1*	Cytochrome P450, family 2, subfamily e, polypeptide 1	-	2.0	1.6	2.3
*Cyp4b1*	Cytochrome P450, family 4, subfamily b, polypeptide 1	-	2.8	-	4.2
**Phase II Metabolizing Enzymes:** Carboxylesterases:					
*Ces1g*	Carboxylesterase 1G	-	2.0	1.5	3.2
*Ces2c*	Carboxylesterase 2C	-	2.3	1.5	2.9
Decarboxylases:					
*Gad1*	Glutamate decarboxylase 1	-	-	-	-
*Gad2*	Glutamic acid decarboxylase 2	-	-	-	-
Dehydrogenases:					
*Adh1*	Alcohol dehydrogenase 1 (class I)	-	1.7	-	1.5
*Adh4*	Alcohol dehydrogenase 4 (class II), pi polypeptide	-	-	-	-
*Adh5*	Alcohol dehydrogenase 5 (class III), chi polypeptide	-	1.5	-	1.6
*Alad*	Aminolevulinate, delta-, dehydratase	-	-	-	-
*Aldh1a1*	Aldehyde dehydrogenase family 1, subfamily A1	-	-	-	-
*Hsd17b1*	Hydroxysteroid (17-beta) dehydrogenase 1	1.7	1.6	-	-
*Hsd17b2*	Hydroxysteroid (17-beta) dehydrogenase 2	-	-	-	-
*Hsd17b3*	Hydroxysteroid (17-beta) dehydrogenase 3	-	-1.9	-	-1.7
Glutathione Peroxidases:					
*Gpx1*	Glutathione peroxidase 1	-	-	-	-
*Gpx2*	Glutathione peroxidase 2	-	-	-	-
*Gpx3*	Glutathione peroxidase 3	-	-	-	-
*Gpx5*	Glutathione peroxidase 5	-	-	-	-
*Gsta1*	Glutathione S-transferase, alpha 1 (Ya)	-	4.6	-	8.9
*Gsta3*	Glutathione S-transferase, alpha 3	-	-	-	2.0
*Gsta4*	Glutathione S-transferase, alpha 4	-	3.0	-	3.3
*Gstm1*	Glutathione S-transferase, mu 1	-	1.7	-	3.8
*Gstm2*	Glutathione S-transferase, mu 2	-	-	-	1.5
*Gstm3*	Glutathione S-transferase, mu 3	-	-	1.8	4.0
*Gstm4*	Glutathione S-transferase, mu 4	-	-	-	1.8
*Gstm5*	Glutathione S-transferase, mu 5	-	-	-	-
*Gstp1*	Glutathione S-transferase, pi 1	-	-	-	-1.5
*Gstt1*	Glutathione S-transferase, teta 1	-	1.9	-	2.7
*Gstz1*	Glutathione S-transferase, zeta 1	-	1.7	-	1.9
*Lpo*	Lactoperoxidase	-	-	-	-
*Mpo*	Myeloperoxidase	-	-53.2	-	-21.6
Hydrolases:					
*Ephx1*	Epoxide hydrolase 1, microsomal	-	2.5	-	4.6
*Faah*	Fatty acid amide hydrolase	-	3.1	-	3.5
*Fbp1*	Fructose bisphosphatase 1	-	-	-	-
Kinases:					
*Hk2*	Hexokinase 2	-1.7	-4.0	-2.4	-2.8
*Pklr*	Pyruvate kinase liver and red blood cell	-	2.7	-	3.4
*Pkm2*	Pyruvate kinase M2	-1.5	-5.3	-2.1	-2.7
Lipoxygenases:					
*Alox12* ^b^	Arachidonate 12-lipoxygenase	-1.4	-5.9	-4.0	-2.6
*Alox15* ^b^	Arachidonate 15-lipoxygenase	-6.7	-	-24.4	-
*Alox5*	Arachidonate 5-lipoxygenase	-	-	-	-
*Apoe*	Apolipoprotein E	-	-	-	-
Oxidoreductases:					
*Blvra*	Biliverdin reductase A	-	-	-	-
*Blvrb*	Biliverdin reductase B (flavin reductase (NADPH))	-	-	-	-
*Cyb5r3*	Cytochrome b5 reductase 3	-	-	-	-
*Gpx1*	Glutathione peroxidase 1	-	-	-	-
*Gpx2*	Glutathione peroxidase 2	-	-	-	-
*Gsr*	Glutathione reductase	-	-	-	-
*Mthfr*	5,10-methylenetetrahydrofolate reductase	-1.9	-2.9	-2.3	-2.2
*Nos3*	Nitric oxide synthase 3	-	1.5	-	1.5
***Nqo1***	NAD(P)H dehydrogenase, quinone 1	1.6	-	1.7	2.0
*Srd5a1*	Steroid 5 alpha-reductase 1	-	3.5	-	2.7
*Srd5a2*	Steroid 5 alpha-reductase 2	-	-	-	-
Paraoxonases:					
*Pon1*	Paraoxonase 1	-	2.2	-	2.3
*Pon2*	Paraoxonase 2	-	-	-	-
*Pon3*	Paraoxonase 3	-	-	-	-
Glutathione S-Transferases:					
*Chst1*	Carbohydrate (keratan sulfate Gal-6) sulfotransferase 1	-	-	-	-
*Gsta3*	Glutathione S-transferase, alpha 3	-	-	-	2.0
*Gstm2*	Glutathione S-transferase, mu 2	-	-	-	-
*Gstm3*	Glutathione S-transferase, mu 3	-	-	1.8	4.0
*Gstm5*	Glutathione S-transferase, mu 5	-	-	-	-
*Gstp1*	Glutathione S-transferase, pi 1	-	-	-	-
*Gstt1*	Glutathione S-transferase, teta 1	-	1.9	-	2.7
*Mgst1*	Microsomal glutathione S-transferase 1	-	-	-	-
*Mgst2*	Microsomal glutathione S-transferase 2	-	-	-	-
*Mgst3*	Microsomal glutathione S-transferase 3	-	2.4	-	2.8
Transferases:					
*Nat1*	N-acetyl transferase 1	-	-	-	-
*Nat2*	N-acetyl transferase 2	-	-	-	-
*Comt*	Catechol-O-methyltransferase	-	-	-	-
*Ggt1*	Gamma-glutamyl-transferase 1	-	-	-	-
Other Genes Related to Drug Metabolism:					
*Abp1*	Amiloride binding protein 1 (amine oxidase, copper-containing)	-	-	-	-
*Ahr*	Aryl-hydrocarbon receptor	-	-	-	-
*Arnt*	Aryl hydrocarbon receptor nuclear translocator	-	-	-	-
*Asna1*	ArsA arsenite transporter, ATP-binding, homolog 1 (bacterial)	-	-	-	-
*Gckr*	Glucokinase regulatory protein	-	1.5	-	1.6
*Marcks*	Myristoylated alanine rich protein kinase C substrate	-	-	-	1.8
*Smarcal1*	SWI/SNF related matrix associated, actin dependent regulator of chromatin, subfamily a-like	-	-	-	-
*Snn*	Stannin	-	-	-	-

Genes were sorted by Functional Gene Grouping according to Mouse Drug Metabolism RT^2^ Profiler^™^ PCR Array (PAMM-002, Qiagen). Expression levels of genes highlighted in boldface were confirmed by TaqMan real-time PCR analysis or were present in both arrays (Table 2, S1 Fig).

^a^These data mean that the gene’s expression is relatively low (threshold cycle is relatively high (C_t_>33) in either the control or the test sample and reasonably detected (C_t_<30) in the other sample suggesting that the actual fold-change value is at least as large as the calculated and reported fold-change result.

^b^This gene’s average threshold cycle is relatively high (C_t_>30), relative expression level is low, in both control and test samples.

### Gene expression of I phase metabolizing enzymes

All the CYP genes were induced by the antioxidants at 7 days in the mouse liver, except for *Cyp17a1*, which was downregulated 4.3-fold by tBHQ and 8.1-fold by TS-13.

Among the analyzed cytochromes, only *Cyp2b10* was already upregulated at 12 h of exposure to both phenolic antioxidants. In addition, expression of *Cyp1a1*, *Cyp2c29*, and *Cyp2e* changed 1.5- to 2.1-fold after 12-h exposure to TS-13 (Table 1). mRNAs of *Cyp27b1* and *Cyp19a1* were not detectable both in control and test samples.

### Gene expression of II/III phase metabolizing enzymes/transporters

The Nrf2-regulated genes of phase II xenobiotic-metabolizing enzymes were upregulated during exposure to antioxidants tBHQ or TS-13 (Table 1). Upregulation of genes of antioxidant defense like *Nqo1*, *Gsta1*, *Gstm1*, *Gstt1*, *Mgst3*, and *Gstz1* was mainly more prominent with TS-13 as compared to tBHQ. Furthermore, we identified additional genes upregulated by TS-13—*Gsta3*, *Gstm3*, *Gstm4*, *Gstm2*, and *Mgst2—*which were not affected by tBHQ in the experimental conditions. *Gsta1* was the most upregulated gene both during tBHQ treatment (4.6-fold) and TS-13 treatment (8.6-fold). Other transferases such as *Nat1/2*, *Comt*, *Ggt1*, and *Gstm5* were not affected by tBHQ or TS-13. Seven days of exposure to tBHQ or TS-13 led to an increase (2.0- to 4.6-fold) in the mRNA expression of hydrolases (*Ephx1*, *Faah*) and carboxylesterases (*Ces1g*, *Ces2c*). The effect of TS-13 was also stronger. Expression of dehydrogenases *Adh1* and *Adh5* was slightly and equally changed after 7 days of treatment with tBHQ or TS-13.

Among other genes related to II phase xenobiotic-metabolizing enzymes, *Hk2* and *Pkm* were downregulated already at 12 h of exposure to tBHQ (1.7- and 1.5-fold, respectively), continuing to decrease by 7 days of treatment (4- and 5.3-fold). For TS-13 this effect was less pronounced (at 12 h by 2.4- and 2.1-fold, respectively, and a 2.8- and 2.7-fold decrease after 7 days). In addition, four genes of phase II enzymes—*Mthfr*, *Alox12*, *Alox15*, and *Mpo—*which are not directly regulated by Nrf2, were downregulated in the liver of mice treated with the synthetic antioxidants. These genes are NFkB targets rather than Nrf2 targets related to an inflammatory response. One of the most downregulated by either tBHQ or TS-13 was *Mpo* (a 53.2- and 21.6-fold change, respectively). Mpo is a known marker of inflammation and converts numerous substrates to reactive free radicals [39].

None of the analyzed peroxidase genes were affected in the liver by the tBHQ or TS-13 administration. The Nrf2 target genes of selenoproteins—*Gpx1*, *Gpx2*, and *Gpx3—*are special cases because their expression depends on selenium status, and *Gpx5* is epididymis specific.

Other genes related to the drug metabolizing system, those encoding transporters, were also affected. mRNA levels of several ATP-binding cassettes (*Abcb1a*, *Abcb2b*, and *Abcb4*) did not vary during treatment with tBHQ or TS-13, and changed by ~2-fold.

The expression levels of two genes *(Mt2* and *Mt3)* involved in protection against heavy metal- and ROS-induced toxicity were affected differently. *Mt3* was upregulated at 12 h, but was not changed after 7 days of treatment. In contrast, the *Mt2* mRNA level was reduced strongly by treatment with tBHQ (12.5-fold) or TS-13 (>20-fold) after 7 days, but just like *Mt3* expression, increased slightly at 12 h of the antioxidants exposure. Little is known about the mechanism underlying the regulation of *Mt2* and *Mt3* gene expression in the mouse liver. It should be mentioned that *Mt3* is mainly expressed in mouse lungs and brain but is still detectable in the liver. Previously, differential regulation of these two genes by nutrition was also shown, and it is thought that they likely have different functions [40].

### Protein expression and an enzymatic activity of CYPs

**Fig 3:**
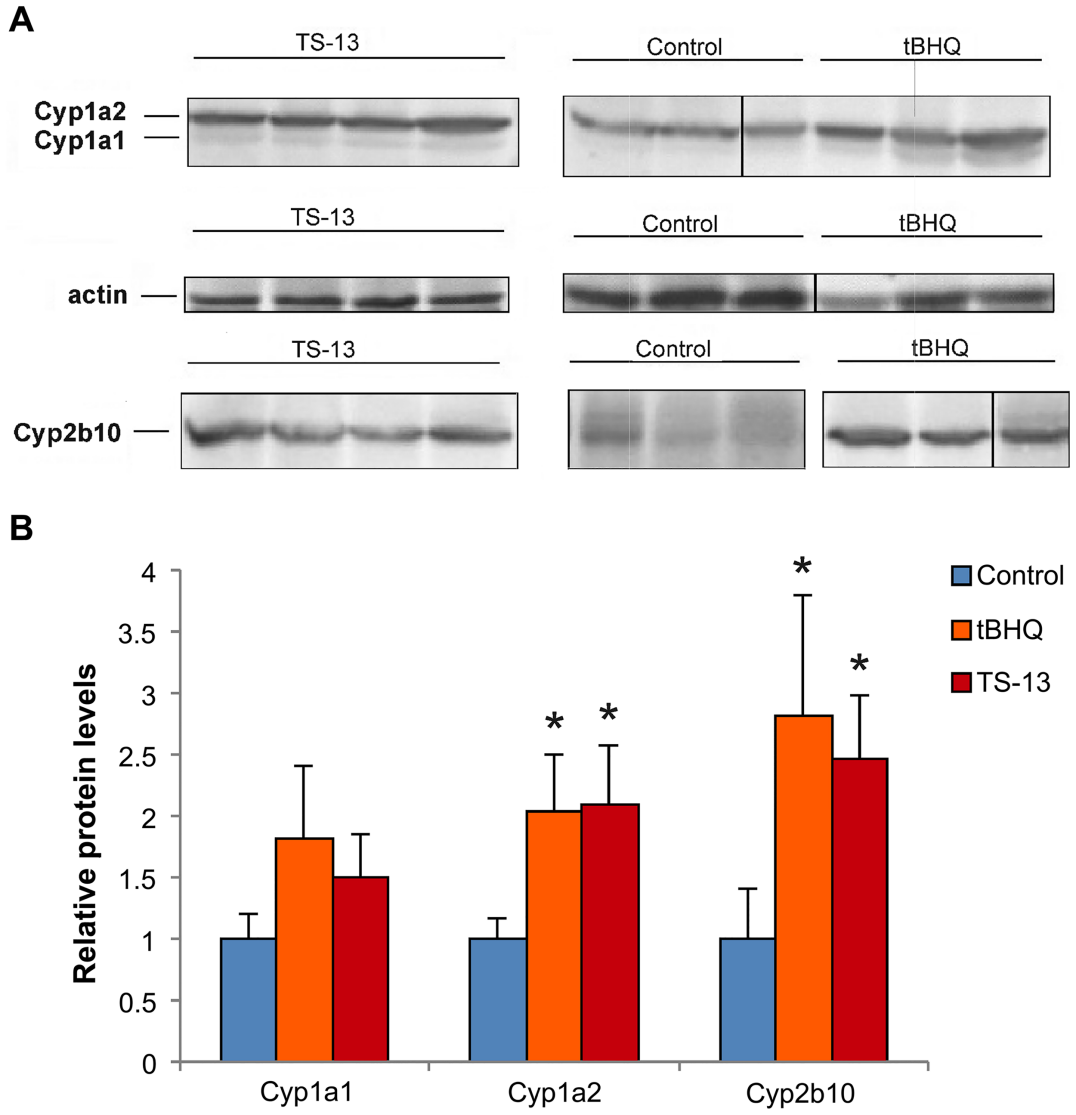
Cyp1a1, Cyp1a2, and Cyp2b protein expression changes after 7 days of tBHQ or TS-13 treatment. (A) Western blot analysis. The number of animals in control and tBHQ groups was 3; in the TS-13 group– 4. (B) Data were densitometrically analyzed and normalized to β-actin. The results are shown as mean ± SD; *p < 0.05.

Western blot and enzyme activity assays were carried out to detect changes in their protein levels and activities. Western blots revealed similar effects of tBHQ and TS-13 on subfamily members Cyp1a1, Cyp1a2, and Cyp2b. tBHQ and TS-13 after 7 days of administration induced a increase (~1.5–2.8-fold) in protein levels of Cyp1a1, Cyp1a2, and Cyp2b. For Cyp1a2 and Cyp2b the changes were significant (Fig 3A and 3B).

**Fig 4:**
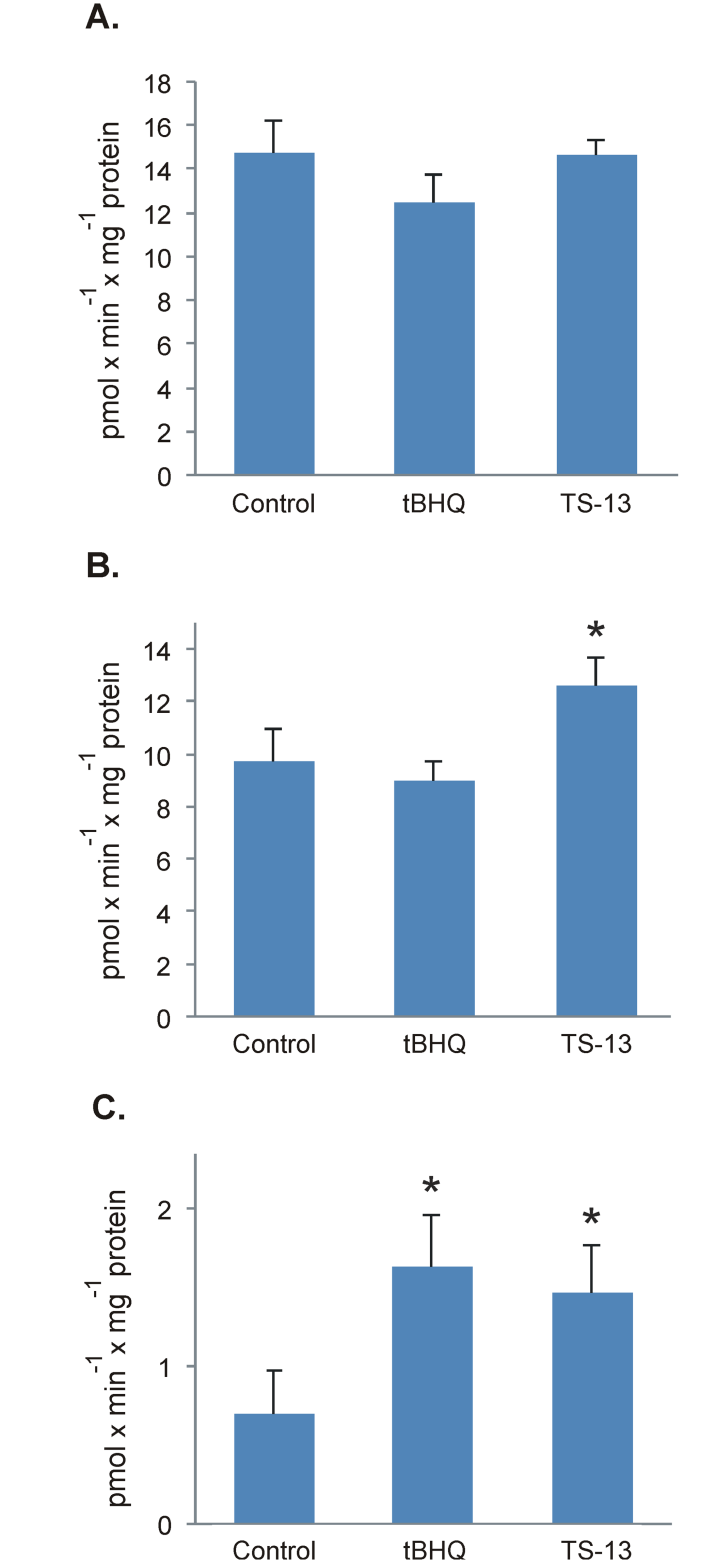
Effects of tBHQ and TS-13 on Cyp1a1 (A), Cyp1a2 (B), and Cyp2b (C) enzymatic activities in pmol × min^-1^ × mg^-1^ protein; *p < 0.05.

Additionally, after 7 days of administration the compounds (tBHQ or TS-13), we observed a significant increase in selective enzymatic activity of Cyp2b in the mouse liver related to a control group (Fig 4C). As for Cyp1a2, a significant increase was observed only with TS-13 (Fig 4B). There were no differences in the activity of Cyp1a1 between the control group and tBHQ or TS-13 groups (Fig 4A).

TBHQ or TS-13 caused changes not only in gene expression (Table 1) but also in the protein levels and enzymatic activities of Cyp1a2 and Cyp2b. This indicates that the changes in the P450 activity are regulated at the transcriptional level.

### Gene expression analysis of signal transduction pathways

In order to establish the regulatory pathways influenced by tBHQ and TS-13, we carried out a real-time PCR array analysis of hepatic mRNA expression using Mouse Signal Transduction PathwayFinder PCR Array including 84 genes related to the main signaling pathways. This analysis revealed that expression of 41 genes was changed by tBHQ at 7 days of treatment and expression of 42 genes was affected by TS-13 (after 7 days of treatment) in the liver of mice (either a >1.5-fold increase or decrease; Fig 2).

Of these 41 and 42 genes, 24 were upregulated, whereas 17 were downregulated by tBHQ, and 27 up- and 15 down-regulated by TS-13, respectively. These gene products participate in a variety of processes, including the oxidative stress response, detoxification, inflammation, proliferation, apoptosis/cell survival, sex steroid signaling or are associated with energy metabolism. Changes in the expression levels of most genes of Signal Transduction PathwayFinder array—as in the case of drug metabolism genes—were time-dependent for tBHQ and TS-13 (Fig 2, Table 2). We detected a large number of changes in gene expression of NFκB, androgen, estrogen, retinoic acid, phosphatidylinositol 3-kinase/ alpha serine-threonine protein kinase (PI3K/AKT), phospoholipase C, wingless-related MMTV integration site (Wnt), and Hedgehog pathways (Table 2, S3 Fig).

**Table 2:** Mouse signal transduction PathwayFinder PCR array.

Gene symbol	Description	tBHQ fold change		TS-13 fold change	
		12 h	7 d	12 h	7 d
**Mitogenic Pathway:**					
*Egr1*	Early growth response 1	-	-7.1	-	-3.3
*Fos*	FBJ osteosarcoma oncogene	-	2.0	-	2.0
***Jun***	Jun oncogene	1.8	-	1.6	
*Nab2*	Ngfi-A binding protein 2	-	-	-	-
**Wnt Pathway:**					
*Birc5*	Baculoviral IAP repeat-containing 5	-	-2.0	-	-1.7
*Ccnd1*	Cyclin D1	-	-2.7	-3.6	-3.2
*Cdh1*	Cadherin 1	-2.2	-2.4	-1.5	-1.6
*Fgf4* ^a^	Fibroblast growth factor 4	3.8	15.0	3.4	12.8
***Jun***	Jun oncogene	1.8	-	1.6	-
*Lef1*	Lymphoid enhancer binding factor 1	-	-	-	-
***Myc***	Myelocytomatosis oncogene	-	-3.3	-	-
*Pparg*	Peroxisome proliferator activated receptor gamma	-	-	2.0	2.0
*Tcf7*	Transcription factor 7, T-cell specific	-	-	-	-
*Vegfa*	Vascular endothelial growth factor A	-	-	-	-
*Wisp1*	WNT1 inducible signaling pathway protein 1	-	1.7	-	3.4
**Hedgehog Pathway:**					
*Bmp2*	Bone morphogenetic protein 2	-	6.5	-	6.9
*Bmp4*	Bone morphogenetic protein 4	-	2.7	-	4.3
*En1*^b^	Engrailed 1	2.5	8.9	3.4	20.6
*Foxa2*	Forkhead box A2	-	-	-	-
*Hhip*	Hedgehog-interacting protein	1.5	4.1	1.8	5.7
*Ptch1*	Patched homolog 1	-	-	-	-
*Wnt1* ^b^	Wingless-related MMTV integration site 1	-	4.6	-	9.1
*Wnt2*	Wingless-related MMTV integration site 2	-	4.9	-	5.3
**TGF-β Pathway:**					
*Cdkn1a*	Cyclin-dependent kinase inhibitor 1A (P21)	-2.7	-3.2	-2.3	-2.2
*Cdkn1b*	Cyclin-dependent kinase inhibitor 1B	-	1.7	-	2.3
*Cdkn2a*	Cyclin-dependent kinase inhibitor 2A	-	-	-	-
*Cdkn2b*	Cyclin-dependent kinase inhibitor 2B (p15, inhibits CDK4)	1.5	2.2	1.5	3.3
**p53 Pathway:**					
*Bax*	Bcl2-associated X protein	-	-	-	-
*Cdkn1a*	Cyclin-dependent kinase inhibitor 1A (P21)	-	-3.2	-2.3	-2.2
*Ei24*	Etoposide induced 2.4 mRNA	-	-	-	-
*Fas*	Fas (TNF receptor superfamily member 6)	-	-2.7	-	-2.9
*Gadd45a*	Growth arrest and DNA-damage-inducible 45 alpha	-	-	-	-
*Igfbp3*	Insulin-like growth factor binding protein 3	2.1	10.1	2.0	21.8
*Mdm2*	Transformed mouse 3T3 cell double minute 2	-	-	-	-
**NFκB Pathway:**					
*Ccl20*	Chemokine (C-C motif) ligand 20	-	-	-	-
*Cxcl1*	Chemokine (C-X-C motif) ligand 1	-	-19.8	-2.5	-10.9
*Icam1*	Intercellular adhesion molecule 1	-1.5	-2.3	-1.5	-1.9
*Ikbkb*	Inhibitor of kappaB kinase beta	-	-	-	-
*Il1a*	Interleukin 1 alpha	-	-	-	-
*Il2*	Interleukin 2	-	-	-	-
*Lta* ^a^	Lymphotoxin A	2.3	10.4	1.6	12.0
*Nfkbia*	Nuclear factor of kappa light polypeptide gene enhancer in B-cells inhibitor, alpha	-	-	-	-
*Nos2*	Nitric oxide synthase 2, inducible	-	-	-	-
*Tank*	TRAF family member-associated Nf-kappa B activator	-	-	-	-
*Tnf*	Tumor necrosis factor	-	-3.0	-	-1.9
*Vcam1*	Vascular cell adhesion molecule 1	-	-1.9	-	-
**NFAT Pathway:**					
*Cd5*	CD5 antigen	-	-	-	-
*Fasl*	Fas ligand (TNF superfamily, member 6)	-	-	-	-
*Il2*	Interleukin 2	-	-	-	-
**CREB Pathway:**					
*Cyp19a1*	Cytochrome P450, family 19, subfamily a, polypeptide 1	-	-	-	-
*Egr1*	Early growth response 1	-	-7.1	-	-3.3
*Fos*	FBJ osteosarcoma oncogene	-	2.0	-	2.0
**Jak-Stat Pathway:**					
*Cxcl9*	Chemokine (C-X-C motif) ligand 9	-1.5	-23.2	-4.0	-12.2
*Il4ra*	Interleukin 4 receptor, alpha	-	-	-	-
*Irf1*	Interferon regulatory factor 1	-2.4	-3.0	-2.2	-2.5
*Mmp10*	Matrix metallopeptidase 10	-	-	-	-
*Nos2*	Nitric oxide synthase 2, inducible	-	-	-	-
**Estrogen Pathway:**					
*Bcl2*	B-cell leukemia/lymphoma 2	-	2.4	-	3.4
*Brca1*	Breast cancer 1	1.9	5.5	1.6	2.0
*Greb1*^a^	Gene regulated by estrogen in breast cancer protein	3.0	7.7	3.8	7.1
*Igfbp4*	Insulin-like growth factor binding protein 4	-	-	-	-
*Nrip1*	Nuclear receptor interacting protein 1	-	1.6	-	1.5
**Androgen Pathway:**					
*Cdk2*	Cyclin-dependent kinase 2	-	1.5	-	1.7
*Cdkn1a*	Cyclin-dependent kinase inhibitor 1A (P21)	-2.7	-3.2	-2.3	-2.2
*Pmepa1*^a^	Prostate transmembrane protein, androgen induced 1	2.2	9.5	2.0	6.5
**Calcium and Protein Kinase C Pathway:**					
*Csf2*	Colony stimulating factor 2 (granulocyte-macrophage)	-	-	-	-
*Fos*	FBJ osteosarcoma oncogene	-	2.0	-	2.0
*Il2*	Interleukin 2	-	-	-	-
*Il2ra*	Interleukin 2 receptor, alpha chain	-	-	-	-
***Jun***	Jun oncogene	1.8	-	1.6	-
*Myc*	Myelocytomatosis oncogene	-	-3.3	-	-
*Odc1*	Ornithine decarboxylase, structural 1	-	-	-	-
*Tfrc*	Transferrin receptor	-	-	-	-
**Phospholipase C Pathway:**					
*Bcl2*	B-cell leukemia/lymphoma 2	-	2.4	-	3.4
*Egr1*	Early growth response 1	-	-7.1	-	-3.3
*Fos*	FBJ osteosarcoma oncogene	-	2.0	-	2.0
*Icam1*	Intercellular adhesion molecule 1	-1.5	-2.3	-1.5	-1.9
***Jun***	Jun oncogene	1.8	-	1.6	-
*Nos2*	Nitric oxide synthase 2, inducible	-	-	-	-
*Ptgs2*	Prostaglandin-endoperoxide synthase 2	2.1	9.9	1.9	13.6
*Vcam1*	Vascular cell adhesion molecule 1	-	-1.9	-	-
**Insulin Pathway:**					
*Cebpb*	CCAAT/enhancer binding protein (C/EBP), beta	-	-	-	-
*Fasn*	Fatty acid synthase	-	-1.5	-2.0	-1.5
*Gys1*	Glycogen synthase 1, muscle	-	-	-	-
*Hk2*	Hexokinase 2	-1.5	-3.0	-1.9	-2.9
*Lep*^b^	Leptin	3.0	3.6	2.0	4.0
**LDL Pathway:**					
*Ccl2*	Chemokine (C-C motif) ligand 2	-1.5	-2.8	-2.08	-2.7
*Csf2*	Colony stimulating factor 2 (granulocyte-macrophage)	-	-	-	-
*Sele*	Selectin, endothelial cell	-	-	-	-
*Selp*	Selectin, platelet	-	-	-	-
*Vcam1*	Vascular cell adhesion molecule 1	-	-1.9	-	-
**Retinoic Acid Pathway:**					
*En1*^b^	Engrailed 1	2.5	8.9	3.4	20.6
*Hoxa1* ^b^	Homeobox A1	1.8	3.2	1.7	5.0
*Rbp1*	Retinol binding protein 1, cellular	-	3.6	-	3.7
**Stress Pathway:**					
*Atf2*	Activating transcription factor 2	-	-	-	-
*Fos*	FBJ osteosarcoma oncogene	-	2.0	-	2.0
*Hsf1*	Heat shock factor 1	-	-	-	-
*Hspb1*	Heat shock protein 1	-	-1.5	-	-1.9
*Myc*	Myelocytomatosis oncogene	-	-3.3	-	-
*Trp53*	Transformation related protein 53	-	-	-	-
**Survival Pathways:** PI3 Kinase / AKT Pathway:					
*Bcl2*	B-cell leukemia/lymphoma 2	-	2.4	-	3.4
*Ccnd1*	Cyclin D1	-	-2.7	-3.6	-3.2
*Fn1*	Fibronectin 1	-	-	-	-
***Jun***	Jun oncogene	1.8	-	1.4	-
*Mmp7*	Matrix metallopeptidase 7	-	2.2	2.2	3.3
***Myc***	Myelocytomatosis oncogene	-	-3.3	-	-
Jak / Src Pathway:					
*Bcl2*	B-cell leukemia/lymphoma 2	-	2.4	-	3.4
*Bcl2l1*	Bcl2-like 1	-	-	-	-
NFκB Pathway:					
*Naip1*	NLR family, apoptosis inhibitory protein 1	-	2.7	-	2.7
*Birc2*	Baculoviral IAP repeat-containing 2	-	-	-	-
*Birc3*	Baculoviral IAP repeat-containing 3	-	-	-	-
*Tert*	Telomerase reverse transcriptase	-	-	-	-

Genes were sorted by signal transduction pathways according to Signal Transduction PathwayFinder RT^2^ Profiler PCR Array description (PAMM-014, Qigene). Some genes were included in several pathways. Expression levels of genes indicated in bold were confirmed by TaqMan real-time PCR (S4 Fig) or are present in both arrays.

^a^These data mean that the gene’s expression is relatively low (threshold cycle is relatively high, C_t_ > 33) in either the control or the test sample and reasonably detected (C_t_ < 30) in the other sample, suggesting that the actual fold change value is at least as large as the calculated and reported fold change result.

^b^This gene’s average threshold cycle is relatively high (C_t_ > 30), relative expression level is low, in both control and test samples.

### Calcium and protein kinase C Pathway

The *Jun* oncogene was upregulated slightly only at 12 h, whereas the *Fos* oncogene was upregulated 2-fold after 7 days of exposure to tBHQ or TS-13. *Myc* expression decreased 3.3-fold after 7 days of tBHQ exposure.

### Phospholipase C Pathway

*Egr1* and *Icam1* were downregulated by both compounds and *Vcam1* only by tBHQ, after 7 days. *Bcl2* and *Fos* were upregulated 2.0–3.4 folds by tBHQ or TS-13 by 7 days of treatment. A mild increase by TBHQ or TS-13 treatment at 12 h was observed for *Jun*. *Ptgs2* was increased by tBHQ or TS-13 (9.9-fold and 13.6-fold, respectively) after 7 days, (at 12 h by both compounds ~2-fold).

### Insulin Pathway

*Fasn* and *Hk2* were both decreased (1.5–2.9 folds) after 7 days of tBHQ or TS-13 treatment. Both genes were affected at 12 h by treatment with TS-13 (~2-fold downregulation). The upregulated gene related to this pathway during treatment with tBHQ and TS-13 was *Lep* (2.0–4.0 folds). *Lep* is expressed mainly in white adipose tissue. The C_t_ value of control mice for *Lep* in the liver was ~34, but in mice treated with tBHQ or TS-13, it was relatively high (C_t_ > 30).

### Retinoic Acid Pathway

Genes *En1* and *Hoxa1* were upregulated both at 12 h and after 7 days of treatment with tBHQ or TS-13, whereas *Rbp1* was upregulated only after a week of the exposure (Table 2). After 7 days of the antioxidant treatment, TS-13 was found to be a stronger inducer of all genes in question: *En1*, *Hoxa1*, and *Rbp1* (20.6-, 5-, and 4-fold, respectively) than tBHQ was (8.9-, 3.2-, and 3.6-fold, respectively).

### Wnt Pathway

Most genes of Wnt signaling were affected by treatment with tBHQ or TS-13, except for *Vegfa*, *Lef1*, and *Tcf7*. Genes *Birc5*, *Ccnd1*, *Cdh1*, and *Myc* were downregulated by tBHQ or TS-13, whereas *Fgf4*, *Wisp1*, and *Pparg* were upregulated (Table 2). *Jun* mRNA expression was affected only after 12 h of tBHQ or TS-13 exposure.

### Hedgehog Pathway

According to the qPCR array results, 6 genes of this pathway were upregulated in the mouse liver by the tBHQ or TS-13 treatment, while *Ptch1* and *Foxa2* were unaffected (Table 2). Expression levels of *Bmp2*, *Bmp4*, *En1*, *Hhip*, *Wnt1*, and *Wnt2* increased after 7 days of treatment with tBHQ or TS-13. In fact, except for *Wnt2*, the upregulation in the case of 7-day treatment with TS-13 was stronger than that with tBHQ. After 12 h of the antioxidant exposure, expression levels of *En1* and *Wnt1* were already increased. Both genes were more affected than other genes after 7 days of treatment.

### NFkB related genes

We should mention the presence of NFkB-regulated genes included in both arrays. *Pstg2*, *Bmp-2*, *Bmp-4*, *Fos*, and *Jun* were upregulated tBHQ or TS-13 (Table 2). However, more NFkB related genes such as *Mthfr*, *Alox12*, *Alox15*, *Mpo*, *Gstp1*, *Egr-1*, *Ccl2*, *Icam*, *Vcam*, *Tnf*, *Cxcl1*, *Cxl9*, *Myc*, *Cdkn1a*, *Ccnd1*, and *Fas* were downregulated (Tables 1 and 2). Some of them were downregulated already after 12 h, showing inhibition of NFkB rather than activation.

Modulation by tBHQ and TS-13, directly or indirectly, of the NFkB pathway probably depends on the tissue type, dose, and time of exposure. As a prooxidant, tBHQ first may cause nonspecific activation of redox sensitive NFkB, and then via activation of Nrf2, MAP cascades (PI3K/Akt which influence the NFkB pathway), and probably direct specific inactivation of NFkB decreases expression of proinflammatory genes.

### Genes related to sex steroids

An intriguing category of genes modulated by tBHQ and TS-13 treatment is related to the sex hormone biosynthesis, metabolism-related genes, such as *Cyp2b10*, *Cyp17a1*, *Srd5a1*, *Hsd17b1*, and *Hsd17b3* (Table 1), genes related to the androgen pathway: *Cdkn1a*, *Pmepa1*, and *Cdk2* (Table 2). The strongest response among genes in the androgen pathway was observed for *Pmepa1* (7 days of tBHQ: 9.5-fold and 6.5-fold for TS-13). Genes of the estrogen pathway, which were included in Signal Transduction PathwayFinder Array—*Bcl2*, *Brca1*, *Greb1*, and *Nrip1* (Table 2)—were also affected. One of the most upregulated genes in this pathway was *Greb1* (7 days of tBHQ: 7.7-fold and 7.1-fold for TS-13).

In summary, we determined that expression of 32 (19%) and 43 (~26%) of all the genes studied (168) was changed after 12-h treatment with tBHQ or TS-13, respectively. Seven days of treatment of BALB/c mice with tBHQ led to the expression changes of 76 genes (~45%; 51 upregulated and 25 downregulated) and with TS-13: 84 genes (50%, 60 upregulated, 24 downregulated; Fig 2).

The study showed that tBHQ and TS-13 had a time-dependent effect on most of the genes under study. It should also be noted that more genes of xenobiotic metabolism were upregulated by TS-13 than by tBHQ. It should be noted that the significance of the response was different. Most of the drug metabolism genes and genes associated with signal transduction pathways responded more strongly to TS-13 exposure than to tBHQ.

## Discussion

As expected from earlier studies [7, 8, 16, 41], we observed a predictable effect of antioxidants tBHQ and TS-13 on Nrf2-regulated genes. Most of the genes of phase II xenobiotic-metabolizing enzymes containing ARE (*Nqo1*, *Gsta1*, *Gstm1*, *Gstt1*, *Mgst3*, *Gstz*, *Gsta3*, *Gstm3*, *Gstm4*, *Gstm2*, *Mgst2*, *Ces1g*, *Ces2c*, *Ephx1*, *Faah*, *Adh1*, and *Adh5*) were upregulated by tBHQ or TS-13 treatment (Table 1). These observations confirm that the presence of ARE in the promoter of multiple detoxification genes results in coordinated induction of these protective enzymes in response to pro-oxidants or electrophiles such as tBHQ [7, 41]. Previously, we have shown that TS-13 also induces Nrf2-regulated genes via ARE activation [16].

The expression of other phase II drug-metabolizing genes, whose products may increase oxidative stress and inflammation by themselves, *Mpo* and *Alox 12/15*, was decreased during tBHQ or TS-13 treatment. These enzymes metabolize their substrates thus causing formation of reactive free radicals and induce oxidative stress and inflammation [39, 42]. This mechanism may be related to induction of AMP-activated protein kinase (AMPK), which is known to inhibit at least *Alox15* expression [43]. In hepatocytes, tBHQ activates AMPK [11].

The *Mthfr* gene as well as *Alox12/15* was found to be downregulated. These genes are NFkB targets rather than Nrf2 targets [44].

In general, both compounds had similar effects on the gene expression of phase II xenobiotic-metabolizing enzymes, showing up- or downregulation of the same genes (Table 1). Nevertheless, in the case of TS-13, more genes were identified as upregulated, and strength of the effect was higher as compared with tBHQ.

Some of the genes that were upregulated in the study include phase I drug metabolism cytochrome P450 among others. The CYP superfamily of microsomal hemoproteins is involved in the metabolism of ~ 90% chemicals (general chemicals, natural, and physiological compounds, and drugs). CYP1A1, 1A2, 1B1, 2A6, 2C9, 2E1, 2B6, and 3A4 contribute greatly to the metabolism of substances in each of these categories [45]. This situation points to a possible significant contribution of CYP enzymes to many of the currently described effects of tBHQ on metabolism or cellular function.

Here we showed that tBHQ and TS-13 increase the gene expression of phase I drug metabolizing enzymes *Cyp1a1*, *Cyp1a2*, *Cyp2b10*, *Cyp2c29*, *Cyp2e1*, *Cyp4b1*, and *Cyp11b2* in the mouse liver. For *Cyp1a1*, *Cyp1a2*, and *Cyp2b10*, we showed that this activation was also manifested in protein levels and activities. P450 induction by synthetic phenolic antioxidants has been observed previously [20–22]. In some studies [20, 21], the researchers proposed that tBHQ induces *Cyp1a1* as a ligand of AhR. Here we also observed upregulation of *Cyp1a1* and *Cyp1a2*, which contain binding sites for AhR. The AhR pathway is known to be involved in the induction of both phase I and II xenobiotic-metabolizing enzymes [46]. The link between AhR and Nrf2 is probably mediated by multiple mechanisms in a species- and tissue-specific manner: Nrf2 is a target gene for AhR, and Nrf2 can be activated by ROS generated by the AhR target Cyp1a1. A direct cross-interaction between AhR and Nrf2 has also been proposed in the regulation of some target genes such as *Nqo1* [47]. In the present work, we also showed an increase in the expression of a target gene of constitutively activated receptor (CAR): *Cyp2c29* (mouse homolog of human *CYP2C19*) during treatment with tBHQ or TS-13. Previously it was shown in human hepatocytes that upregulation of other phase I xenobiotic-metabolizing enzymes such as CYP2C19 and CYP2C9 is mediated by distinct activation of the AP-1 site in the promoters of these genes. It has been shown elsewhere, using specific kinase inhibitors, that extracellular signal-regulated kinase (ERK) and c-Jun N-terminal kinase (JNK) are essential for the tBHQ-induced expression, whereas Nrf2 has no effect [22]. We also observed induction of mRNA, protein expression, and activity of Cyp2b10, another CAR target gene (Table 1, Figs 3 and 4C). Previously, it was shown that BHA, a metabolic precursor of tBHQ, induces Cyp2b10 4.9-fold [23]. Therefore, activation of CAR should be considered another potential mechanism by which the phenolic antioxidants may induce P450s. Of note, it has been hypothesized that the majority of CAR activators work through an indirect dephosphorylation pathway, just as phenobarbital does, instead of binding directly to CAR [48]. Induction of CYP1A and CYP2B points to activation of AhR and CAR pathways, respectively [49]. Simultaneous interaction of tBHQ (or TS-13) with AhR and CAR as a ligand is unlikely because the ligand-receptor interactions are distinguished by strict structural constraints. Prototypipical AhR ligands (TCDD and benzopyrene) do not bind to CAR, and the prototypipical CAR ligand, 1,4-Bis[2-(3,5-dichloropyridyloxy)]benzene [50], does not bind to AhR [51]. Therefore, the involvement of other regulatory factors in the observed response to the antioxidants is more likely.

CYP1A1/A2, and 2B are known to bioactivate compounds by turning them into toxic or carcinogenic forms, in addition to detoxifying other compounds [24, 52]. Therefore, the promotion of chemical carcinogenesis by tBHQ [3–5, 20] (and probably by TS-13) in some organs may be linked to this mechanism). We found that the mRNA levels of most genes of phase I enzymes present in Mouse drug metabolism arrays were elevated by tBHQ or TS-13, except for *Cyp17a1*, which was downregulated (Table 1). Cyp17a1 has both 17α-hydroxylase and 17,20-lyase activities and is a key enzyme in the steroidogenic pathway that produces progestins, mineralocorticoids, glucocorticoids, androgens, and estrogens. Serum testosterone levels decrease, as do gene expression and activity of *Cyp17a1* in the testes of mice treated with 100 mg/kg tBHQ for 7 days [53]. We also have demonstrated earlier that administration of TS-13 for 2, 4, or 7 days decreases *Gstp1* expression in the liver of male mice [34]. Here, we also observed the decrease in gene expression (Table 1). It is known that mouse *Gstp1* is expressed predominantly in the male liver and is regulated by androgens. The promoter of murine *Gstp1* contains 7 binding sites for androgen receptor (AR), and 3 AREs for Nrf2 [54]. The gene expression changes may be explained the possible change in the serum testosterone level, as shown elsewhere [53].

Transporter genes were also found to be transcriptionally affected by tBHQ or TS-13 in the mouse liver. Phase III transporters play crucial roles in drug absorption, distribution, and excretion. Along with phase I and phase II enzymes, phase III transporters are thought to be similarly regulated in a coordinated fashion, and provides an important mechanism of protection of the body from xenobiotic exposure.

Taken together, our results suggest that tBHQ or TS-13 may coordinately regulate genes of phase I, II, and III xenobiotic-metabolizing enzymes/transporters via Nrf2-dependent pathways as well as Nrf2-independent signaling. The regulation of these genes may have significant preventive effects on tumor initiation by enhancing the cellular defense system, preventing the activation of procarcinogens/reactive intermediates, and by increasing the excretion/efflux of reactive carcinogens or metabolites. Carcinogenic activity of tBHQ (or TS-13), at least in part may be related to the activation of CYPs as well as Nrf2 because of its dual role in carcinogenesis [55].

The extent of cross-talk between all the phase I, II, and III regulators increases the complexity of the molecular interaction that needs to be elucidated and means that individual effects of phenolic antioxidants tBHQ and TS-13 on the oxidative status of the cell, on receptors, on transcriptional factors, and protein kinases are difficult to distinguish.

Further we studied the influence of tBHQ and TS-13 on gene expression modulated by various cellular signals. We uncovered changes in the expression of a large number of genes associated with NFkB, androgen, estrogen, retinoic acid, PI3K/Akt, stress, Wnt, and Hedgehog pathways (Table 2 and S2 Fig).

Previous other studies have revealed that Nrf2 inducers suppress NFkB-related proinflammatory genes [29]. The actual mechanisms via which tBHQ reduces gene expression of proinflammatory factors have yet to be fully elucidated though evidence suggests that NFkB may be involved (directly or indirectly) in the regulation of the expression of proinflammatory genes. In one study, it was shown that tBHQ markedly decreases NFκB activation, production of inflammatory cytokines, and ICAM-1 expression in the gut [27]. NFkB dependent genes were shown to be modulated by tBHQ in Jurkat cells. tBHQ inhibits IL-2 and CD25 expression, which correlates with decreased NFκB transcriptional activity in the cell [56]. As for treatment with TS-13, also has an anti-inflammation activity [33], we shown downregulation of p50 and p65 subunits of NFkB [34].

At the same time, other studies in vitro revealed that tBHQ activates NFkB, thereby increasing the expression of its target genes [57]. Probably this opposite effect depends on the dose, duration, and timing of antioxidant exposure as well as redox status, and the type of cell. Moreover, both activating and inhibitory effects of ROS on NFkB are known [58].

Indeed, in our study, we observed downregulation of NFkB target genes related to inflammation (*Mthfr*, *Alox12*, *Alox15*, *Mpo*, *Gstp1*, *Egr-1*, *Ccl2*, *Icam*, *Vcam*, *Tnf*, *Cxcl1*, *Cxl9*, *Myc*, *Cdkn1a*, *Ccnd1*, and *Fas*) during tBHQ or TS-13 treatment, showing implying inhibition rather than activation of NFkB.

In addition, expression of *Egr1* was found to decrease after 7 days of tBHQ or TS-13 treatment (Table 2). Egr1 usually acts in synergy with NFkB for the activation of transcription of proinflammatory mediators but can also itself modulate NFkB activity transcriptionally and post-transcriptionally via direct interaction with an NFkB subunit or indirectly by inducing an activator of NFkB, such as tumor necrosis factor alpha (TNF-α) [59] (whose gene expression was decreased in our study, Table 2).

Taken together, the observed inhibitory effects of tBHQ and TS-13 on genes related to inflammation may be due to the suppression of NFkB pathways and activation of Nrf2 as well as the influence on kinases involved in the Nrf2-NFkB crosstalk [32] though additional research is needed to identify the mechanisms behind these possible relations.

The mechanism underlying modulation of Nrf-2 and NFkB-regulated genes by tBHQ (or TS-13) may be related to activation of PI3K/Akt signaling. PI3K controls nuclear translocation of Nrf2, mediates Nrf2 activation, and thus contributes to induction of antioxidant phase II enzymes via coordinated gene transactivation [60, 61]. Induction of *GSTA2* and *NQO1* expression by tBHQ is mediated by PI3K/Akt signaling: inhibitors of PI3K/Akt prevent binding of Nrf2 to ARE and block *GSTA2* and *NQO1* expression [62, 63]. In our study, genes directly regulated by this pathway were also affected: *Bcl2* expression increased and *Ccnd1* expression decreased.

In addition, PI3K/Akt signaling has numerous crosswalks with other pathways. In our study, tBHQ and TS-13 activated genes of Hedgehog signaling (Table 2, S2 Fig). The Hedgehog pathway performs key functions in embryonic development and tumorigenesis. In one study, it was demonstrated that the Hedgehog signaling pathway is activated during various processes related to liver injury, including liver regeneration [64]. Several studies show that PI3K/Akt signaling activates this pathway [65, 66].

Activation of Wnt signaling may upregulate its target genes *Ccnd1* and *Myc*, which were downregulated in our study (Table 2). Components of Wnt signaling as well as Hedgehog regulate tissue proliferation and regeneration. These two signaling networks are not isolated and interact with each other and other pathways to coordinate proliferation and regeneration. Moreover, the Wnt pathway is known to be sensitive to cellular redox status and can be triggered by mild ROS. Toxic liver injury upregulates Wnt signaling components. In addition, it is thought that Nrf-2 activation may inhibit Wnt signaling [67, 68].

AR may be modulated by phosphorylation in the absence of androgens either directly or indirectly by the MAPK cascade and other signaling pathways that contribute to modulation of AR gene expression. Activation of the PI3K/Akt pathway also results in phosphorylation of AR, suppression of AR target genes, such as *p21* (*Cdkna1*), and a decrease in androgen/AR-mediated apoptosis [69]. Indeed, in our study, we detected a decrease in expression of *Cdkna1* and other genes regulated by AR *Cyp17a1* and *Ccnd1*. *Cyp17a1* expression is negatively regulated by AR. By contrast, *Greb1*, a direct target of the AR, was upregulated. On the other hand, this gene may also be regulated by estrogen receptor (ER), and one study also showed *Greb1* to be a novel target gene of Nrf2 [70], which means that the increased gene expression may be related to activation of Nrf2. Other genes related to sex steroids, such as *Srd5a1*, *Gstp1*, *Cyp2b10*, *Hsd17b1*, *Hsd17b3* (Table 1), *Pmepa1*, *Bcl2*, *Brca1*, and *Nrip1* (Table 2) were also upregulated. At least for *Srd5a1*, it is known that mRNA is upregulated by Akt activation [71].

A recent study on mouse hepatocytes and human hepatoma cells indicates that tBHQ induces autophagy in an Nrf2-independent manner via activation of AMPK [11]. It is also known that tBHQ markedly reduces ATP concentration [72, 73]; as a result, activation of AMPK, a cellular energy sensor responding to low ATP levels, triggers signaling pathways that replenish cellular ATP supplies. AMPK is also known to promote anti-inflammatory responses. This phenomenon is mediated by inhibition of NFκB and stimulation of Akt [74].

Once activated, AMPK mediates switch cells from active ATP consumption (fatty-acid and cholesterol synthesis) to active ATP production (oxidation of fatty acids and glucose). The signaling cascades initiated by the activation of AMPK affect expression of genes of glucose and lipid metabolism, inhibit lipogenesis, and improve insulin sensitivity. These effects are crucial for regulation of metabolic events in the liver.

Indeed, in our study, we observed changes in gene expression of energy metabolism enzymes and factors such as Fasn, Hk, Pklr, Pkm2, Faah, Ephx1, Gckr, Lep, and Igfbp3. Energy metabolic pathways of retinoid and insulin were affected. Other genes—whose products may influence energy metabolism related to androgen and estrogen pathways—were also affected (as discussed above).

In conclusion, synthetic phenolic antioxidants tBHQ and TS-13 have multiple targets in the cell and most likely act via activation Nrf2, inhibition of NFkB pathways, activation of several kinase cascades such as PI3K/Akt and AMPK (and probably others), thereby modulating their complex crosstalk net causing changes in a number of genes related to drug, lipid, insulin, and sex steroid metabolic pathways; to antioxidant defense; regeneration; and inflammation. These latter signaling events are difficult to distinguish from the initial events because of the involvement of the complex crosstalk and multiple feedback and feedforward regulatory mechanisms.

## Supporting information

S1 Fig mRNA expression of drug metabolism enzymes (A) Cyp2b, (B) Nqo1 in the mice liver (n = 5) at 12 h and 7 days after tBHQ or TS-13 treatment relative to control mice. Gene expression was analyzed by real-time qRT-PCR (TaqMan) and normalized to the mean of reference genes *Gapdh* and *b-actin*. *—p < 0.05.
(TIF)

S2 Fig Western blots used to create Fig 3. (TIF)

S3 Fig Effects of 7-days treatment with tBHQ (A) or TS-13 (B) on the expression of genes of different signaling pathways in the mouse liver. Genes that were either induced or repressed over 1.5-fold are listed.
(TIF)

S4 Fig mRNA expression of (A) Myc, (B) Jun genes in the mice liver (n = 5) at 12 h and 7 days after tBHQ or TS-13 treatment relative to untreated mice. Gene expression was analyzed by real-time qRT-PCR (TaqMan) and normalized to the mean of reference genes *Gapdh* and *b-actin*. *—p < 0.05.
(TIF)
