# Supplementary figures and images for: The gene expression profile of a drug metabolism system and signal transduction pathways in the liver of mice treated with tert-butylhydroquinone or 3-(3'-tert-butyl-4'-hydroxyphenyl)propylthiosulfonate of sodium
Source: PLoS One. 2017 May 3;12(5):e0176939. doi: 10.1371/journal.pone.0176939 (PMC5415222; doi:10.1371/journal.pone.0176939)

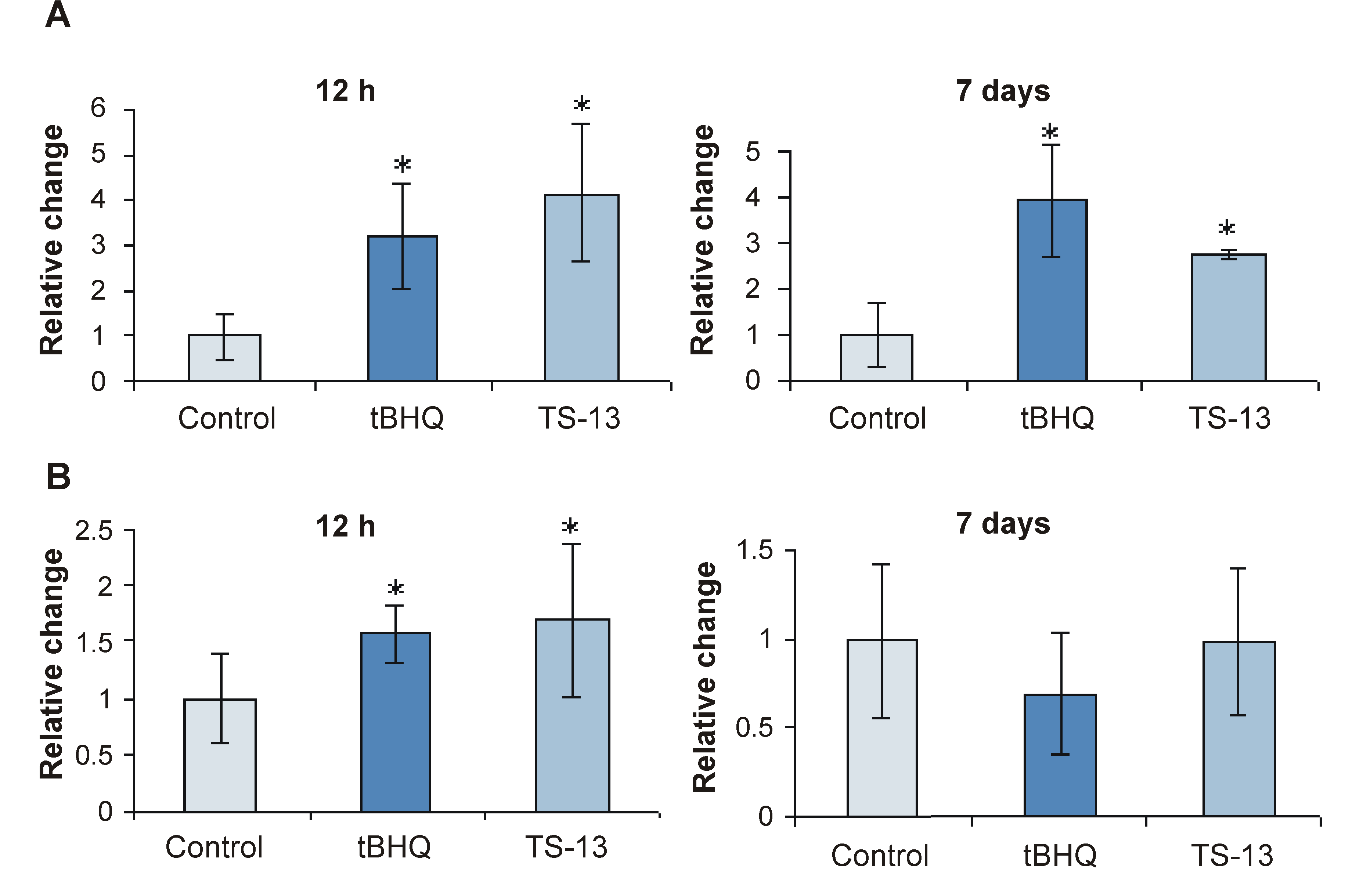

Supplement: S1 Fig — Gene expression was analyzed by real-time qRT-PCR (TaqMan) and normalized to the mean of reference genes Gapdh and b-actin. *—p < 0.05. (TIF) [file pone.0176939.s001.tif]

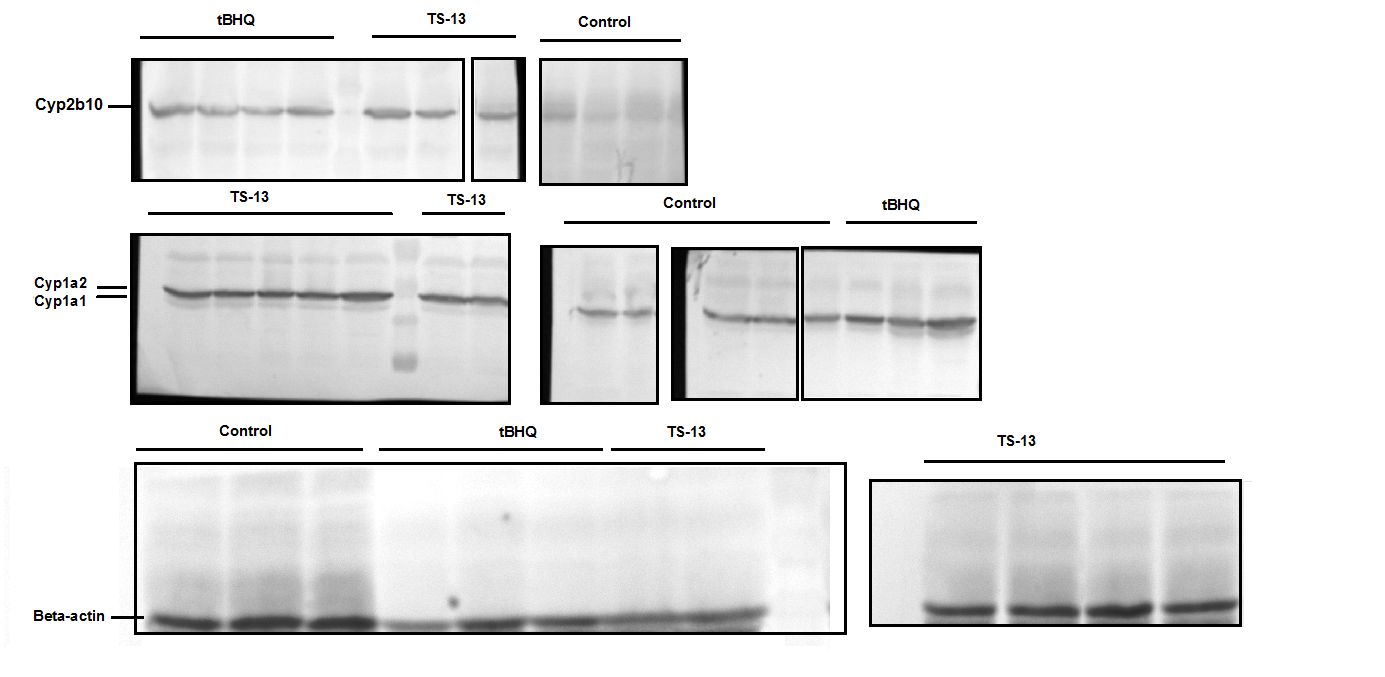

Supplement: S2 Fig — (TIF) [file pone.0176939.s002.tif]

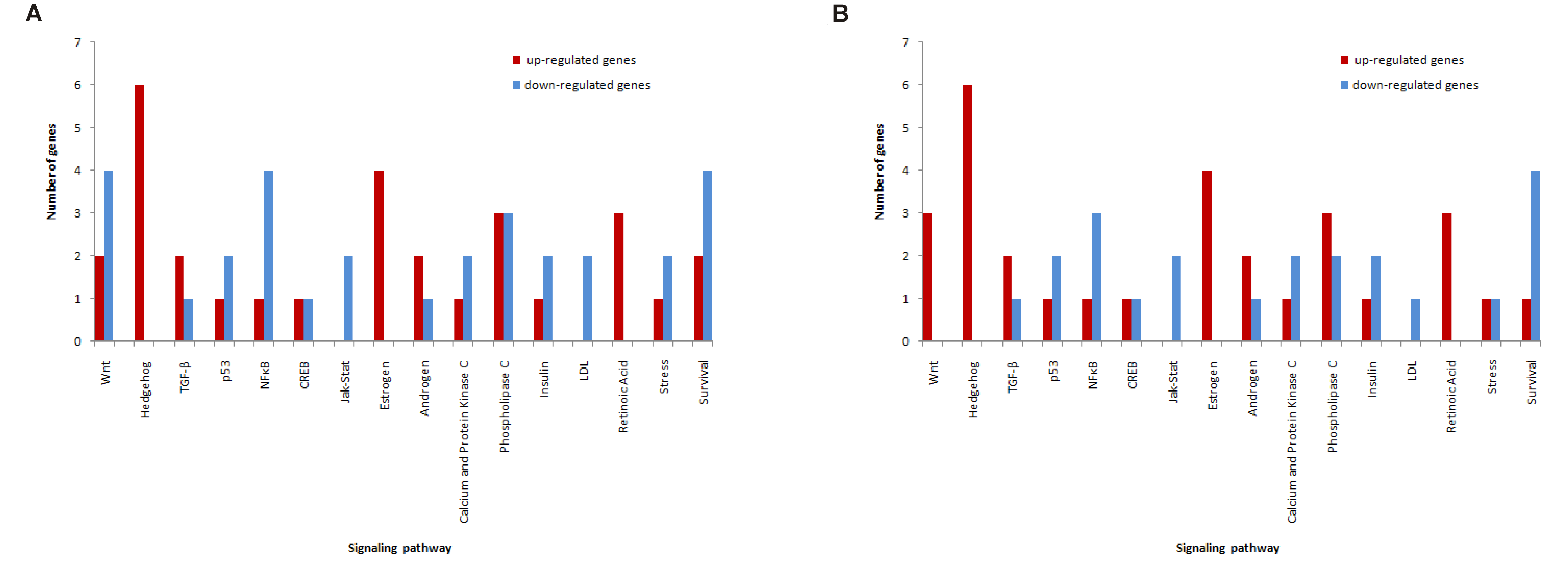

Supplement: S3 Fig — Genes that were either induced or repressed over 1.5-fold are listed. (TIF) [file pone.0176939.s003.tif]

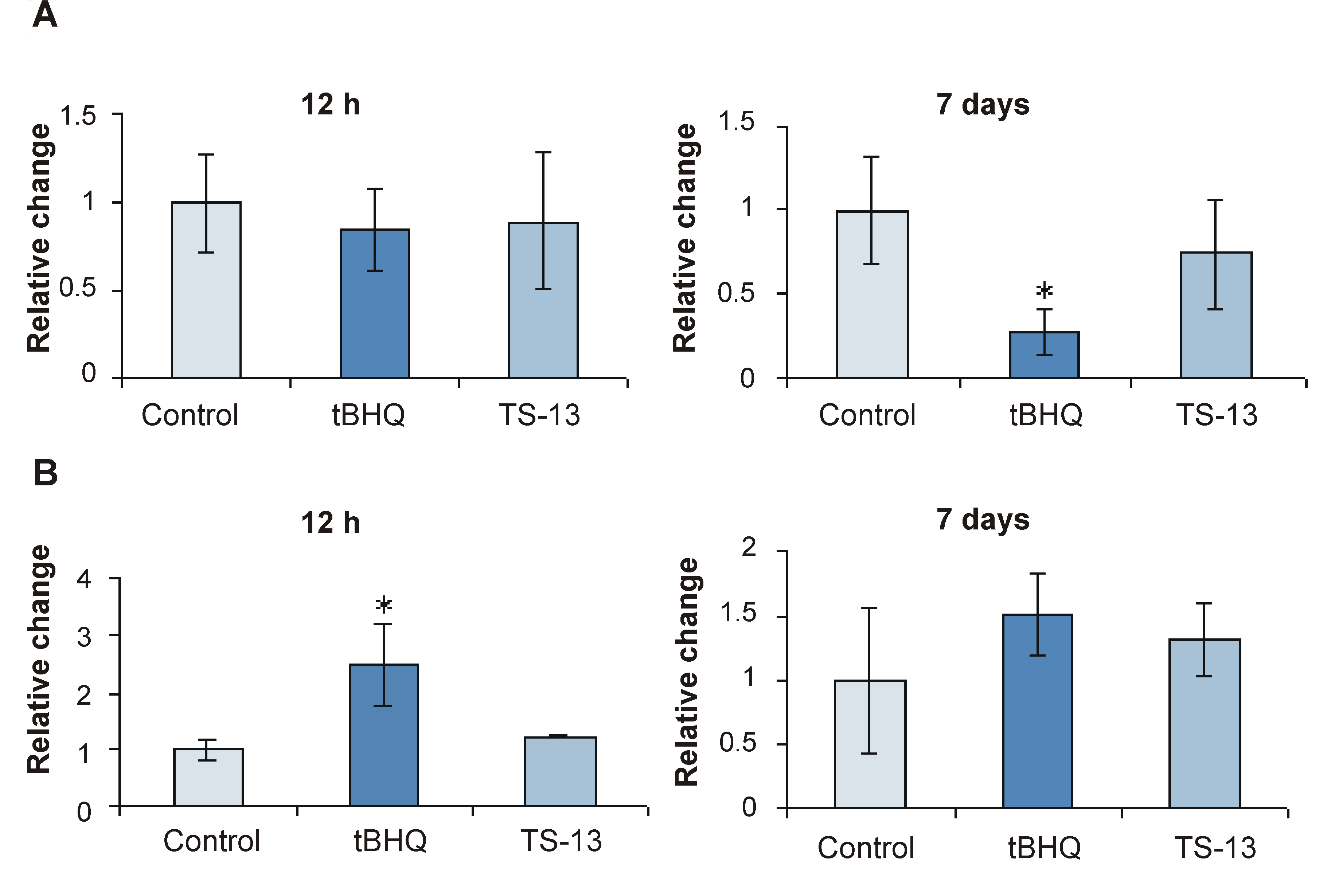

Supplement: S4 Fig — Gene expression was analyzed by real-time qRT-PCR (TaqMan) and normalized to the mean of reference genes Gapdh and b-actin. *—p < 0.05. (TIF) [file pone.0176939.s004.tif]
